# Crowding and Follicular Fate: Spatial Determinants of Follicular Reserve and Activation of Follicular Growth in the Mammalian Ovary

**DOI:** 10.1371/journal.pone.0144099

**Published:** 2015-12-07

**Authors:** Francisco Gaytan, Concepcion Morales, Silvia Leon, David Garcia-Galiano, Juan Roa, Manuel Tena-Sempere

**Affiliations:** 1 Department of Cell Biology, Physiology and Immunology, University of Córdoba, 14004, Córdoba, Spain; 2 CIBER Fisiopatología de la Obesidad y Nutrición, Instituto de Salud Carlos III, 14004, Córdoba, Spain; 3 Instituto Maimónides de Investigación Biomédica (IMIBIC)/Hospital Universitario Reina Sofía, 14004, Córdoba, Spain; 4 Department of Pathology, University of Córdoba, 14004, Córdoba, Spain; 5 FiDiPro Program, Department of Physiology, University of Turku, Kiinamyllynkatu 10, FIN-20520, Turku, Finland; Colorado State University, UNITED STATES

## Abstract

Initiation of growth of resting ovarian follicles is a key phenomenon for providing an adequate number of mature oocytes in each ovulation, while preventing premature exhaustion of primordial follicle reserve during the reproductive lifespan. Resting follicle dynamics strongly suggest that primordial follicles are under constant inhibitory influences, by mechanisms and factors whose nature remains ill defined. In this work, we aimed to assess the influence of spatial determinants, with special attention to clustering patterns and crowding, on the fate of early follicles in the adult mouse and human ovary. To this end, detailed histological and morphometric analyses, targeting resting and early growing follicles, were conducted in ovaries from mice, either wild type (WT) or genetically modified to lack kisspeptin receptor expression (Kiss1r KO), and healthy adult women. Kiss1r KO mice were studied as model of persistent hypogonadotropism and anovulation. Different qualitative and quantitative indices of the patterns of spatial distribution of resting and early growing follicles in the mouse and human ovary, including the Morisita’s index of clustering, were obtained. Our results show that resting primordial follicles display a clear-cut clustered pattern of spatial distribution in adult mouse and human ovaries, and that resting follicle aggrupation is inversely correlated with the proportion of follicles initiating growth and entering into the growing pool. As a whole, our data suggest that resting follicle *crowding*, defined by changes in density and clustered pattern of distribution, is a major determinant of follicular activation and the fate of ovarian reserve. Uneven follicle crowding would constitute the structural counterpart of the major humoral regulators of early follicular growth, with potential implications in ovarian ageing and pathophysiology.

## Introduction

The primordial follicles constitute the basic units of the mammalian ovary. Growing follicles are recruited from the pool of resting primordial follicles, to provide mature eggs for cyclic ovulation. The widely accepted view is that the ovarian reserve is established early, during fetal development or early neonatal period, and is progressively depleted during the reproductive lifespan, leading finally to ovarian senescence [1–3].

Two different, age-dependent patterns of follicle activation exist, corresponding to the pre-pubertal and post-pubertal periods. During fetal development in humans [4], and the first postnatal days in rodents [5–7], primordial follicles located at the medullary zone of the ovary star growing as soon as they are formed. In contrast, cortical primordial follicles remain initially dormant. Recent tracing studies have documented that these two populations of primordial follicles exhibit distinct developmental dynamics [8,9]. In the mouse, the first wave of growing follicles activates simulta-neously and gives rise to a nearly synchronous cohort of growing follicles that reach advanced secondary stage by postnatal day 15 (PND-15), and early antral stage at PND-21. This primordial follicle population is responsible for the first ovulation at puberty and is prevalent during the first two months of age, being progressively replaced by growing follicles derived from the post-pubertal primordial follicle population, from 2–3 months of age onwards [9]. In fact, the adult ovarian reserve derives from cortical primordial follicles that initially remain dormant, from which growing follicles are recruited during adult reproductive lifespan. The size of the pool of resting follicles, which constitutes the adult ovarian reserve, seems to be established during pre-pubertal development. Adjustment of the ovarian reserve appears to occur mainly by primordial follicle attrition [10,11], resulting in adequate numbers of resting primordial follicles at puberty [12].

Since the pool of resting follicles is the source from which growing follicles are recruited during the reproductive lifespan, regulation of the rate of entry into the growing pool is essential to maintain the ovarian reserve and to provide adequate numbers of growing follicles for ovulation. During the last decade, the study of genetically modified mouse models has provided an ever-growing amount of exciting data on the signaling pathways and regulatory factors responsible for dormant follicle activation [13]. For instance, genetic manipulation of the components of key signaling pathways, such as PTEN-PI3K-AKT-FOXO3, has been shown to result in phenotypes characterized by either massive follicle activation or inhibition of the primordial-primary follicle transition, revealing their essential roles in follicle awakening [2,3,13].

Primordial follicles are composed of the oocyte and surrounding pre-granulosa cells. The relative roles of these two components in follicle activation are not fully understood. Follicular activation depends on the expression of oocyte-specific transcription factors such as Lxh8, Sohlh1, Sohlh2, and Nobox [14–16], whereas the granulosa cell-specific transcription factor Foxl2 is needed to initiate follicle growth [17]. Functional interactions between the oocyte and the surrounding pre-granulosa/granulosa cells are necessary for follicle growth, and several signaling systems, such as cKIT/KIT ligand (KL) and GDF-9/BMP15, play central roles in follicle activation [18]. In addition, it has been recently reported that activation of mTORC1 in pre-granulosa cells induces KL secretion and triggers follicle activation [19].

Resting follicle dynamics strongly suggest that primordial follicles are under constant inhibitory influences by systemic and/or local factors. The selective activation of some (few) resting follicles, while the majority of them remain dormant for months or years, strongly suggests that local inhibition is the most likely scenario. Numerous signals, including neurotrophins, cytokines and growth factors, have been identified in the oocyte and/or ovarian somatic cells, which might play a role in this phenomenon. These include KL, platelet-derived growth factor (PDGF), hepatocyte growth factor (HGF), keratinocyte growth factor (KGF), vascular endothelial growth factor (VEGF), nerve growth factor (NGF), glial-derived neutotrophic factor (GDNF), basic fibroblast growth factor (bFGF) and members of the BMP family [2,3,13]. On the other hand, several signals, such as AMH, somatostatin and the cytokine, CXCL12, are known to act as inhibitors of follicle recruitment [3]. Yet the mechanisms responsible for the fate of individual resting follicles, which at any given time can be activated, remain dormant or undergo follicular attrition, are poorly understood. Most likely, different combinations of stimulatory, survival and inhibitory local signals interplay to determine the fate of individual resting follicles. Initial studies suggested that the rate of recruitment of dormant follicles into the growing pool was inversely correlated with the size of the resting follicle pool [20,21]. Recently, a computational study by Da Silva Buttkus and colleagues in neonatal mouse ovaries inferred the existence of local inhibitors of follicle activation derived from surrounding resting follicles and from the ovarian surface epithelium [22]. It must be stressed, however, that during the first postnatal days, most, if not all, growing follicles belong to the first wave of (medullary) growing follicles, and hence observations from this early period might not be representative of regulatory phenomena occurring later in postnatal/reproductive life. Indeed, as activation of primordial follicles of the first follicular wave occurs immediately after formation, without entering into a resting stage, regulatory mechanisms at this early age might be different from those affecting adult primordial follicles. It was therefore unclear if these conclusions could be applicable to the adult resting follicle pool.

In this study, we analyzed the relationship of resting follicle crowding and spatial distribution patterns with the rate of recruitment of follicles into the growing pool in the normal mouse and human cycling ovary. In addition, follicular dynamics and follicular spatial relationships were also studied in the ovaries of a murine model of persistently low gonadotropin secretion and anovulation.

## Methods

### Animals

Female C57BL76 mice (referred hereafter as wild type, WT), and mice of the same genetic background with targeted disruption of the gene *Kiss1r* (a.k.a., *Gpr54)*, encoding the kisspeptin receptor (referred hereafter as Kiss1r KO [23]) were used. Generation, genotyping and housing of Kiss1r KO mice (and their corresponding WT littermates) were performed as described in detail elsewhere [23]. Experimental protocols involving the use of this mouse line were approved by the Córdoba University Ethical Committee of animal experimentation and conducted in accordance with the European Union guidelines for use of experimental animals.

WT animals were euthanized on postnatal day (PND) 21 (n = 6), and at 1- (n = 5), 3- (n = 7), 5- (n = 10), 8- (n = 5) and 12- (n = 5) months of age. Kiss1r KO mice were euthanized at PND-21 (n = 3), and at 3- (n = 10), 5- (n = 8), 8- (n = 3) and 12- (n = 5) months of age. The ovaries were fixed in Bouin fluid and, after dehydration, embedded in paraffin wax. Serial ovarian sections (7 μm-thick) were generated and stained with hematoxylin and eosin.

### Human Samples

Human ovarian samples were obtained from the archives of the Department of Pathology of the University of Cordoba, as in previous studies from our group [24,25]. These samples were collected between 1980 and 2000, in keeping with contemporary legislation and standard clinical practices (including informed consent). All samples were treated anonymously, in keeping with current standards of personal data protection. Due to the time elapsed since collection of the tissues, obtaining permission from the donors for inclusion of the samples in this specific study was not considered feasible. Because of this fact and the nature of the analysis to be applied the samples (involving non-invasive histochemical staining of tissue sections, primarily obtained in the context of pathology analysis), permission from an independent Institutional Review Board was not deemed necessary. In any event, consent for the use of these archival samples was given, on the basis of the above considerations, by responsible members of the Department of Pathology, in keeping with standard operational procedures at our Institution [24,25]. The ovarian specimens were obtained from cycling women undergoing oophorectomy, due to gynecological, non-ovarian pathology, not subjected to hormonal therapy. A total of 34 ovaries were selected for the present study, with the following age range: 17–25 year-old (n = 11), 26–30 year-old (n = 10), 31–35 year-old (n = 7) and 36–40 year-old (n = 6) women. Tissues were fixed in buffered 4% formaldehyde and embedded in paraffin. Sections (7 μm-thick) were stained with hematoxylin and eosin.

### Follicle classification and counting

In mouse ovaries, primordial (defined as the unit formed by the oocyte surrounded by flattened granulosa cells), transitional (oocyte surrounded by a mixture of flattened and cuboidal granulosa cells) and early primary (oocyte surrounded by cuboidal granulosa cells, but lacking oocyte growth and a follicle size smaller than 30-μm in diameter) were globally considered as *resting follicles* (RF). This is in line with previous studies, based on the criteria for follicle classification by Pedersen and Peters [26], where follicle types 2 and 3a are considered as non-growing, which have been conducted in rodents [27] and humans [28–30]. In addition, in a recent tridimensional study by Faire et al. (2015), using immunostaining of the oocyte nucleus for follicle identification, early primary follicles were considered as non-growing, as that study used nuclear volume of the oocyte to read out quiescence and oocyte nucleus diameter does not increase in these early follicles [31]. In fact, there is a wealth of literature that supports that not only primordial but also transitional and early primary follicles are still under strong inhibitory influences and should be considered as non-growing or resting follicles. Indeed, transitional follicles may represent >80% of the small follicle population of the ovary [32,33], and labeling experiments in rodents strongly suggest that transition from primordial to primary follicles is actually an extremely protracted process in which such transition follicles, although committed to grow, actually correspond to resting follicles [33]. In contrast, late primary follicles (growing oocyte and a layer of cuboidal granulosa cells with a follicle diameter ≥ 30 μm) were considered as *early growing follicles* (**Panel A** in **S1 File**). Follicles showing 2 or more layers of granulosa cells and lacking an antrum were considered as *secondary follicles*.

In human ovaries, primordial, transitional and early primary follicles (showing less than 50-μm in diameter and no enlarged oocyte) were classified as *resting follicles*, in keeping with previous studies [28–30], whereas late primary (with a layer of cuboidal granulosa cells, enlarged oocyte and a diameter ≥ 50 μm), as well early secondary (showing 1–2 layers of granulosa cells) were classified as *early growing follicles* (**Panel B** in **S1 File**).

In mouse ovaries, a systematic random sampling procedure was used to determine the number of follicles per ovary. According to this procedure, a section was selected at random among the first 10 ovarian sections, and then every tenth section was scored. The numbers of resting, early growing and secondary follicles were counted; the follicle was scored when the oocyte nucleus (primordial follicles) or the nucleolus (primary and secondary follicles) was present in the section. As these counts correspond to 1/10 of the total follicles, the total counting was multiplied by 10 to obtain an estimate of the total numbers of follicles per ovary. Atretic follicles, which were rarely observed at these follicle stages, were not counted.

In order to estimate, at each age-point analyzed, the probability of small follicles to be actually growing, the total number of early growing follicles per ovary was divided by the number of resting plus early growing follicles (*proportion of growing small follicles*). Preliminary pilot studies following 100 individual follicles in serial sections to analyze the possibility of double counting (namely, how many follicles show the nucleus or nucleolus in two adjacent sections) indicated that a maximum of ~10% of resting follicles could be double counted. As this bias is not differential and affects equally to all groups, we did not use correction factors. On the other hand, the nucleolus that was used as a landmark for counting large primary and secondary follicles was present in two adjacent sections in less than 1% of cases, and therefore double counting was negligible.

### Analysis of follicular crowding

To analyze to what extent the number of small follicles per ovary affects follicle crowding, we determined the percentage of resting follicles that have at least one resting follicle in their close proximity. The threshold distance for considering two resting follicles as neighbors was set at ≤40 μm, as measured with a micrometer eyepiece incorporated to the microscope. Of note, this was the largest distance at which a neighbor effect was found in a previous study in the neonatal ovary [22]. Since ovarian follicles in adult mice are actually not as closely packed as in neonatal ovaries, this 40-μm limit was considered an appropriate, conservative distance for our current analyses in pubertal and adult ovaries. Importantly, this distance corresponds to (approximately) two resting follicle diameters, and is in the range of intercellular communication distances by diffusible signals in other systems. For instance, in the *Drosophila* ovary, Wingless (Wg) signaling promotes follicle stem cell proliferation at a distance of about 50-μm from the Wg source [34]. As general procedure for analysis of resting follicle crowding, we scored all resting follicles present in five non-consecutive, randomly selected ovarian sections in five animals per group, in 1-, 3-, and 5-mo-old WT animals, and 3- and 5-mo-old Kiss1r KO mice.

### Number of neighbors in resting and early growing follicles

To analyze whether the numbers of resting follicle neighbors was different between resting and early growing follicles, we determined the number of neighbors at an inter-follicular distance of 60-μm or less, in 3 month-old WT mice and in human ovaries. Since the aim of this particular analysis was to evaluate the possible influence of resting follicle neighbors on early growing follicles, whose diameter is ≥ 30 μm, we followed the same criterion as the one described above for resting follicle neighbors, and, hence, considered an inter-follicle distance of 60-μm, which corresponds to approximately two (early growing) follicle diameters. This distance was also used for human follicles, which are slightly larger than mouse follicles. Again, this distance was considered compatible with intercellular communica-tion distances by diffusible signals in other systems, such as the Drosophila ovary [34]. In mice, 30–40 early growing follicles per animal were scored in non-consecutive ovarian sections. All resting follicles present in the same ovarian section were also scored. This represents a total number of 161 early growing and 311 randomly selected resting follicles (pooled data).

In human ovaries, due to the lower number of early growing follicles and the limited availability of ovarian samples, a total of 100 early growing follicles were scored for the number of resting follicle neighbors in 20 human ovaries (aged 23 to 37 years, selected by the presence of early growing follicles in the available ovarian tissues). All resting follicles present in adjacent microscopic fields with the x20 objective were also scored. This represents a total number of 100 early growing and 390 randomly selected resting follicles (corresponding to pooled data from the different ovarian samples).

### Analysis of spatial pattern distribution of resting follicles

To analyze if follicle crowding was determined not only by the number (i.e., density) of resting follicles but also by their uneven distribution, we studied the spatial pattern distribution of resting follicles by using the Morisita’s index of clustering [35,36], applying a square plot sampling method (**S1 Fig**). For this, a square lattice incorporated to the microscope was positioned along the ovarian cortex systematically, beginning by a randomly selected central ovarian section and applying plot sampling to non consecutive sections until reaching 50 plot sample units per ovary. Using this approach, the number of resting follicles per sample was counted. At the magnification used, the sampling area corresponded to 200x200 μm for mouse ovaries and 400x400 μm for human ovaries. The Morisita’s index was obtained as I_δ_ = Q [(∑xi^2^ –N)/N(N-1)], where Q corresponds to the number of sample units, xi to the number of follicles in each sample unit, and N = ∑xi. Values greater than 1 indicate a clustered distribution of follicles. The index was obtained for 1-, 3- and 5-mo-old WT mice, and for 3- and 5-mo-old Kiss1r KO mice (n = 5), as well as for the total 34 human ovaries included in the study.

### Statistical analyses

Multiple comparisons among means were performed by ANOVA followed by Student-Newman-Keuls test. Student t-test was applied when two means had to be compared. When relevant, linear regression analyses were conducted using Graph-Pad Prism software, for correlations between (i) the proportion of small growing follicles and the remaining resting follicles, (ii) the percentage of follicles with neighbors and the remaining resting follicles, and (iii) the proportion of small growing follicles and the percentage of follicles with neighbors. Of note, regression analyses were applied only to data from WT animals (blue dots). In addition, 95% confidence intervals (CI) and prediction intervals (PI) for the above regression slopes (of WT data) were calculated using the Prism software, and plotted as blue (CI) and grey (PI) dotted lines. Since the range of variation of some variables (e.g., % of follicles with neighbors) was very narrow in Kiss1r KO, regression analyses were not applied to this set of data. Yet, individual data from Kiss1r KO animals were also plotted as red dots in the corresponding figures. In addition, the association between the presence of close resting follicle neighbors and the follicle status (either resting or early growing) was analyzed by determining the relative probability of growing and the odds ratio in the absence *vs*. presence of resting follicle neighbors.

The significance of the Morisita’s index is given by Z = (I_δ_ – 1) / (2/Qm^2^) ^½^, where m corresponds to the mean. Clustering is statistically significant for Z ≥ 1.96 [37], thus indicating a clustered rather than random spatial pattern distribution.

## Results

### Age-related changes in small follicle populations in WT and Kiss1r KO mice

**Fig 1:**
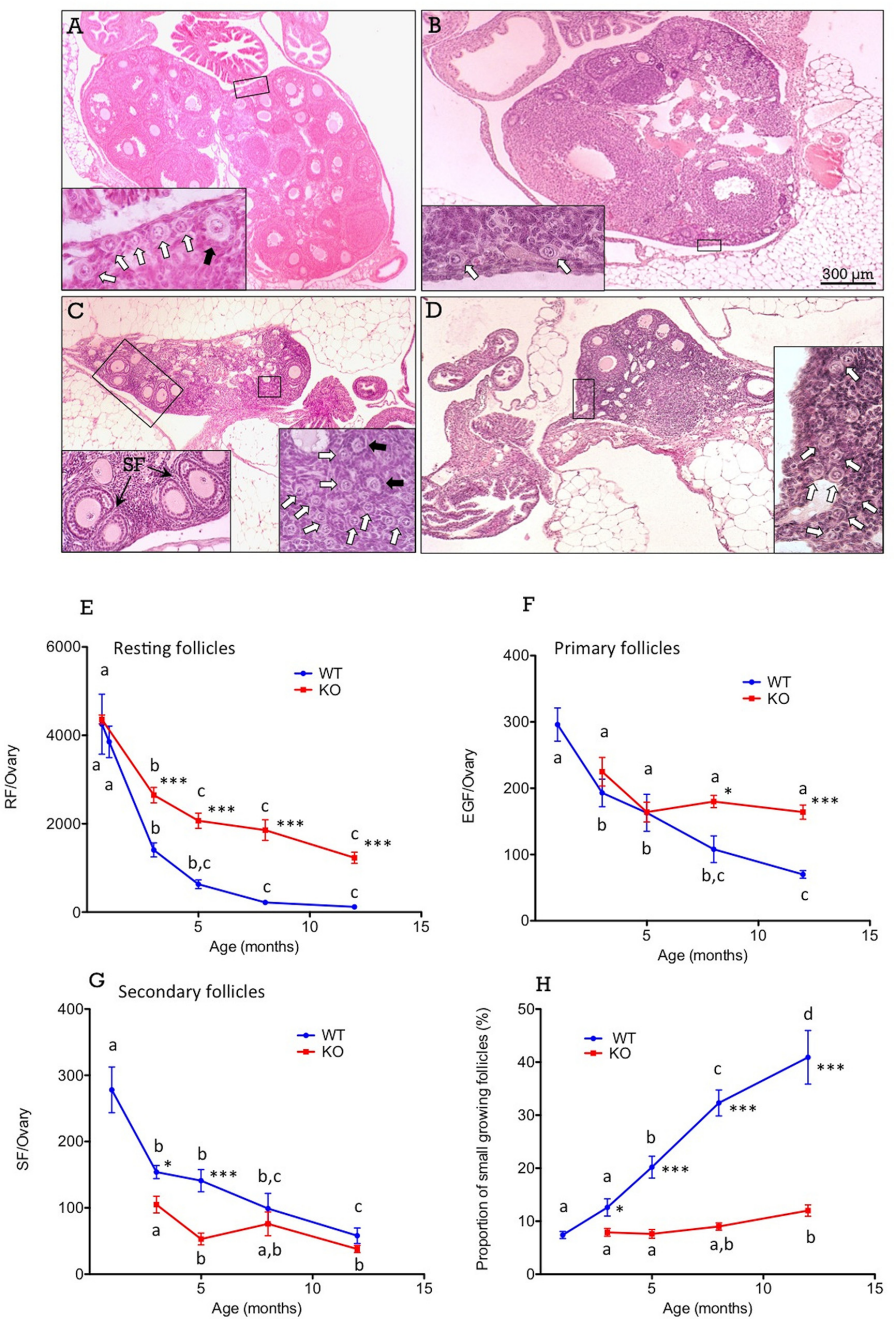
In Panels A-D, representative ovarian sections are presented from WT mice at 1- (A) and 3- (B) months of age, and from Kiss1r KO mice at 3- (C) and 12- (D) months of age. Boxed areas are shown at higher magnification in the insets. Resting (white arrows), primary (black arrows) and secondary (SF) follicles are indicated. In Panels E-H, quantitative analyses of age-related changes are shown for the population of resting (RF; see E), early growing (primary or EGF; see F) and secondary (SF; see G) follicles, and in the proportion of small growing follicles (H) in WT and Kiss1r KO mice. The later (proportion) was calculated by dividing EGF by the total figure of RF+EGF in each ovary. Different superscript letters indicate statistically significant differences between age-points within each genotype (P<0.05 ANOVA followed by Student-Newman-Keuls test); *, P<0.05 vs. corresponding WT values at the same age-point (Student t-test).

Although our study was particularly focused on the adult ovarian reserve, PND-21 WT and Kiss1r KO mice, as well as 1 mo-old WT mice, were also included in our histological analyses in order to obtain baseline data for comparative (time-course) analyses of changes in the number, density and spatial pattern distribution of resting follicles in both genotypes (defined by rather different gonadotropic backgrounds, as Kiss1r KO mice display persistently suppressed gonadotropin secretion [38]). The numbers of resting (i.e., primordial, transitional and early primary), early growing (i.e., large primary) and secondary follicles per ovary were assessed at these and later age-points of postnatal maturation. Representative photomicrographs of ovarian sections from 1-mo and 3-mo-old WT mice, as well as 3-mo and 12-mo-old Kiss1r KO mice are shown in **Fig 1A–1D**. Quantitative analysis of histological data is shown in **Fig 1E–1H**.

In WT mice, the population of resting follicles decreased rapidly from PND21/1-mo (pre-pubertal stage) to 3-months of age, and more slowly from 3- to 12-months of age (**Fig 1E**). The numbers of early growing/primary (**Fig 1F**) and secondary (**Fig 1G**) follicles also decreased with age, but variations were not so rapid, and actually no significant changes in the total numbers of primary and secondary follicles were found from 3- to 5-months of age in WT mice. Kiss1r KO mice showed a number of resting follicles at PND21 equivalent to that of WT, and significantly higher numbers of resting follicles thereafter (**Fig 1E**). The number of early growing/primary follicles in Kiss1r KO was similar to WT animals up to 5-months, but it was significantly higher at 8- and 12-months of age (**Fig 1F**). However, the number of secondary follicles was significantly lower in Kiss1r KO mice at 3- and 5-months of age (**Fig 1E**); an observation that is in agreement with the presence of abundant atretic large secondary follicles in KO animals, in contrast with the seldom observation of such atretic follicles in WT animals.

### Age-related changes in the proportion of early growing follicles in WT and Kiss1r KO mice

The proportion of small, early growing (i.e., primary) follicles with respect to the total number of small (resting and early growing) follicles indicates the probability of resting follicles to initiate growth. In WT mice, this proportion showed a steady increase with age (**Fig 1H**). Whereas a small proportion (about 12%) of small follicles were growing in 3-mo-old WT mice, nearly half (about 41%) of the small follicles were growing at 12-months of age. These figures are in agreement with previous data in rodent [12] and human [39] ovaries. In contrast, in Kiss1r KO mice, the proportion of early growing follicles did not show significant changes from 3- to 8-months of age, and was only slightly increased at 12-months of age (**Fig 1H**); yet, at this age point, the % of small growing follicles in Kiss1r KO mouse ovaries was equivalent to that of WT animals at 3-months of age. The lack of relevant changes in the proportion of early growing follicles in Kiss1r KO mice, which are markedly hypogonadotropic [23], as function of age is compatible with a role of gonadotropins (and eventually local ovarian kisspeptin signaling) in adjusting the probability of resting follicles to initiate growth, while ageing by itself would not play a significant function in this phenomenon.

### Proportion of early growing follicles in relation to the ovarian reserve

**Fig 2:**
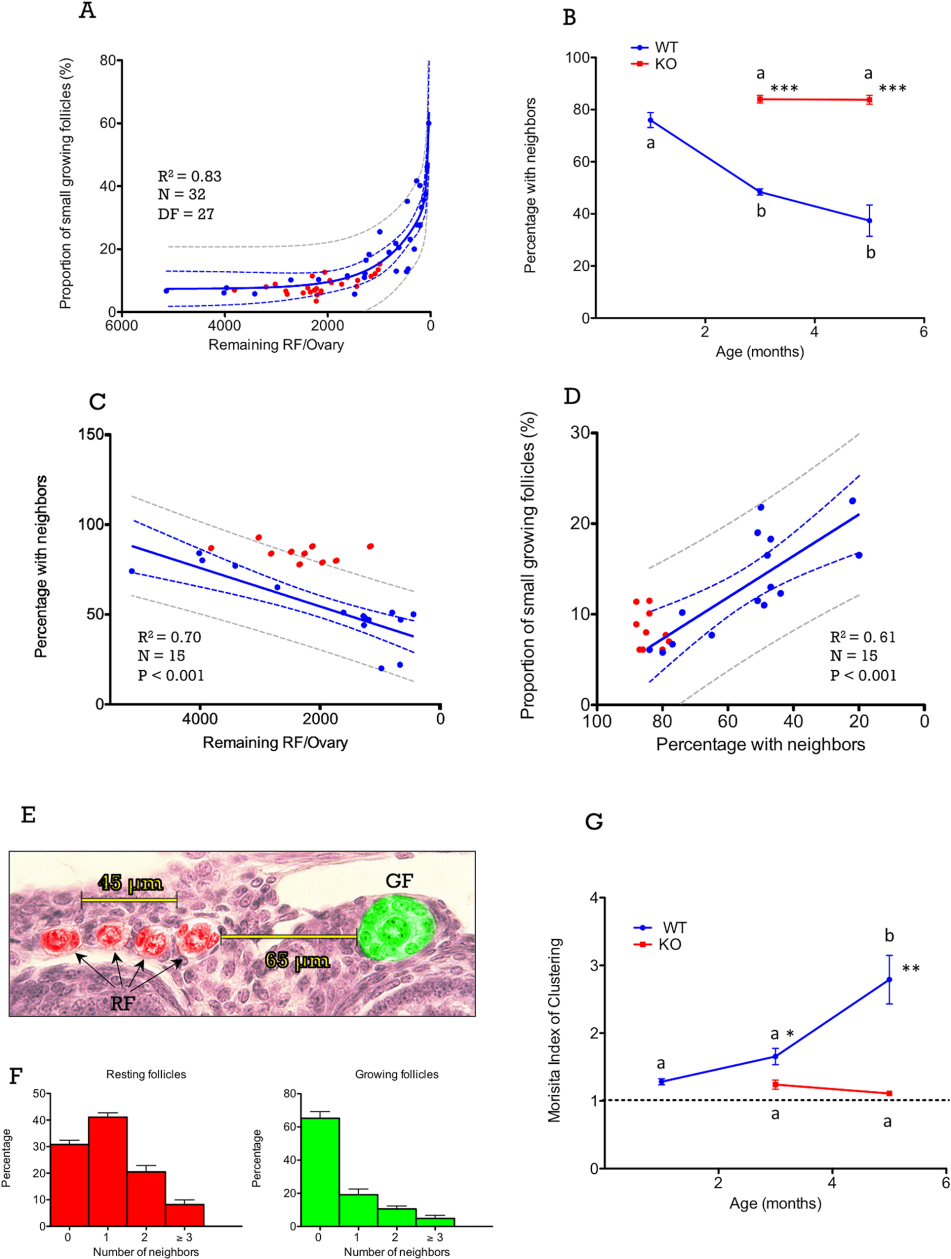
In Panel A, the proportion of small (early) growing follicles is plotted against the number of remaining resting follicles (RF) per ovary. In addition, mean changes in the proportion of resting follicles with at least one close neighbor with respect to age are shown in Panel B, while the proportion of resting follicles (RF) with at least one neighbor are plotted against the number of remaining resting follicles (RF) in Panel C. In addition, in Panel D the correlation between the proportion of early growing follicles and the percentage of follicles with close neighbors is displayed. In all figures, data from WT and Kiss1r KO mice are shown, as blue and red dots, respectively. Yet, regression analyses were applied only to WT data. Similarly, the confidence intervals (CI, presented as blue dotted lines) and the predictive intervals (PI, presented as grey dotted lines) were calculated for the regression slopes obtained from WT individuals. In Panel E, a schematic figure is presented to illustrate of the method used to evaluate the presence of resting follicle neighbors (at a distance of ≤ 60 μm) for resting and early growing follicles. An early growing follicle (GF) lacking close neighbors is highlighted in green, whereas each of the four resting follicles (RF), which have each three neighbors, is marked in red. In Panel F, a distribution plot of frequencies of the number of neighbors in resting and early growing follicles is presented for 3 moth-old WT mice. Data represent the mean ± SEM for n = 5/group. Finally, in Panel G, the Morisita’s index of clustering in WT and Kiss1r KO mice is shown. Values above the dotted line indicate clustered spatial pattern. Different superscript letters indicate statistically significant differences between age-points within each genotype (P<0.05 ANOVA followed by Student-Newman-Keuls test); *, P<0.05 vs. corresponding WT values at the same age-point (Student t-test). Regression analyses, including r^2^ and P values, as well as CI and PI calculations, were conducted using Graph-Pad Prism software. DF: Degrees of freedom.

Initial studies suggested the existence of an inverse correlation between the proportion of follicles leaving the resting pool and the total number of resting follicles in rodent [20,21] and human [39] species. Our present data are also suggestive of an inverse correlation between the probability of a given resting follicle to initiate growth and the magnitude of ovarian reserve. When the proportion of small, early growing follicles was plotted against the remaining pool of resting follicles in WT mice, this proportion increased exponentially along with the decrease of the ovarian reserve, with a sharp rise when the number of resting follicles fell below ~500 resting follicles per ovary, as illustrated by regression analyses shown in **Fig 2A**. Distribution of the proportion of growing follicles vs. the remaining resting follicle pool in Kiss1r KO mice tended to fit the model of WT mice, with values that were in keeping with regression predictions according to their resting follicle population. In fact, all individual values from KO mice fitted within the 95% confidence (CI) and prediction (PI) intervals; the latter represents a probability ≥ 95% of an independent observation (from KO mice) to fall within the regression model defined on the basis of the observed WT data. It must be noted, however, that due to the dynamics of resting follicle pool in the KOs, no individuals with <500 resting follicles per ovary were actually detected. In any event, our regression model suggests that the proportion of early growing follicles is influenced by changes in the total population of resting follicles, irrespective (to a large extent) of the presence or absence of gonadotropins.

### Changes in resting follicle crowding as function of age and the ovarian reserve

If the probability of resting follicles to initiate growth is influenced by changes in the ovarian reserve, this would suggests the existence of a *quorum sensing* mechanism that might mechanistically explain such association. A plausible way for dormant follicles to sense the size and changes of the ovarian reserve is resting follicle density (referred hereafter as *crowding* of resting follicles), which is dependent on the numbers and distribution patterns of resting follicle numbers per ovary. Representative images illustrating changes in density of resting follicles are presented in **Fig 1A–1D**. In WT animals, the density of resting follicles decreased rapidly from 1- to 3-months of age due to both the loss of resting follicles (see also **Fig 1E**) and to the increase of post-pubertal ovarian volume, caused mainly by the appearance of corpora lutea associated to ovulation (**Fig 1A and 1B**). From 3- to 12-months of age, no relevant changes in ovarian volume occurred (*data not shown*) and, hence, the progressive decline in resting follicle density was due mostly to the decrease of the ovarian reserve (see **Fig 1E**). On the other hand, the ovaries of Kiss1r KO mice did not show post-pubertal changes in volume and remained small due to the absence of antral follicles and corpora lutea (see **Fig 1C and 1D**), because of defective gonadotropin drive and anovulation [23]. This feature, together with persistently higher numbers of resting follicles than in WT, determined a higher density of resting follicles in Kiss1r KO mice, from 3- to 12-months of age, evidenced by the presence of large clusters of resting follicles (illustrated in **Fig 1C and 1D**).

Most of the ovarian volume in cycling mice is occupied by corpora lutea, antral follicles, and interstitial tissue, while resting follicles are located in a narrow subsurface area. Hence, it is unclear to what extent changes in the size of the ovarian reserve affect resting follicle crowding. Thus, to obtain a proxy index of resting follicle crowding, we determined changes in the percentage of resting follicles that have at least one close resting follicle neighbor (at 40-μm or less), and analyzed changes in follicle crowding with respect to age and to the size of the ovarian reserve. As shown in **Fig 2B**, the percentage of resting follicles that had at least one resting follicle neighbor in their close proximity significantly decreased from 1- to 5-months of age in WT animals, whereas it did not overtly change between 3- and 5-months in Kiss1r KO mice. Furthermore, a strong correlation between follicle crowding (estimated as the percentage of resting follicles with neighbors) and the size of the ovarian reserve was found in WT animals (see **Fig 2C**), but not in Kiss1r KO mice.

The direct correlation between follicle crowding and the size of the resting follicle pool in WT animals suggests that changes in the ovarian reserve can be sensed through changes in the numbers of nearby resting follicle neighbors. In good agreement, the proportion of early growing follicles (as index of probability of growing) was negatively correlated with resting follicle crowding (see **Fig 2D**), meaning that the probability of growing increases as inverse function of the degree of crowding. When data from Kiss1r KO ovaries were represented against the regression plots of WT animals, they roughly fitted the CI and PI of the regression slope of WT data, suggesting that this phenomenon might not be primarily affected by the gonadotropin input. It must be noted that, because of higher crowding of resting follicles in Kiss1r KO mice, all data from this genotype clustered at the left extreme of the regression curve (i.e., >80% of RF with neighbors).

### Effects of crowding on the probability of initiating growth in resting follicles

If the probability of a resting follicle to initiate growth is influenced by follicle crowding (namely, the proximity of other resting follicles), then small, early growing follicles should lack close neighbors at a higher frequency than resting follicles. To test this hypothesis, we determined the number of neighbors (at a distance of 60-μm or less) in resting and early growing follicles in 3-mo–old WT mice. An illustration of the counting procedure is provided in **Fig 2E**; one growing follicle without resting follicles at <60 μm is marked in green, while different resting follicles with neighbors within this distance are highlighted in red in the same section. Qualitative analyses of these data revealed that whereas most (>70%) resting follicles had at least one close neighbor (see **Fig 2F**; red bars), only 35% of early growing follicles had at least one neighbor (hence ~65% did not have resting follicle neighbors) (see **Fig 2F**; green bars). Such differences in the pattern of distribution are fully compatible with the hypothesis that the presence of resting follicle neighbors in their proximity decreases the probability of activation of follicle growth.

The association between the small follicle status (either resting or growing) and the presence or absence of nearby resting follicle neighbors was further analyzed in 3 month-old WT mice by determining the relative probability and the odds ratio (OR) to initiate growth (**Table 1**). Resting follicles without neighbors have 2.64 times (95% CI: 2.01, 3.45) more probability to initiate growth, and growing follicles have 4.52 times (95% CI: 3.01, 6.78) higher odds of lacking close neighbors. These results document the existence of a significant association between the probability of resting follicles to initiate growth and the absence of other resting follicles in their proximity.

**Table 1:** Association analysis between follicle status and the presence of resting follicle neighbors in 3-mo-old WT mice.

	Growing	Resting	RP (95% CI)	OR (95% CI)
Without neighbors	106	93		
With neighbors	55	218	2.64 (2.01–3.45)	4.52 (3.01–6.78)

RP: relative probability of growing; OR: odds ratio

### Age-related changes in resting follicle clustering in WT and Kiss1r KO mice

Our histological analyses revealed that resting follicles are not evenly distributed through the ovarian cortex, particularly in post-pubertal animals. This feature determines that resting follicle crowding is not only determined by the number of resting follicles (i.e., the size of the ovarian reserve), but also by the pattern of (uneven) distribution of resting follicles in the ovarian cortex. Quantitative measures were used to analyze the spatial pattern distribution of resting follicles in the ovarian cortex to ascertain whether this uneven distribution is or not a random phenomenon. As a method to assess clustering, we used the Morisita’s index of clustering [35,36], which determines whether the spatial pattern of object distribution is uniform, random or clustered. Values greater than 1 indicate a clustered spatial distribution. This index has the advantage of not being affected by changes in object density, which is a relevant issue for our study, since number of resting follicles decreases with age.

Based on our initial observations, we hypothesized that if isolated resting follicles have a higher probability to initiate growth than grouped resting follicles, then the clustering of resting follicles (as indicated by the Morisita’s index) should increase with age in WT mice. In good agreement with this hypothesis, data in **Fig 2G** indicate that the spatial distribution of resting follicles was clustered (Morisita’s index >1) rather random, from 1- to 5-months of age, in WT mice, and clustering significantly increased with age. In contrast, although resting follicles in Kiss1r KO mice also showed some sort of clustered distribution (similar to that seen in 1-mo-old WT mice), the clustering index did not significantly increase with age (see **Fig 2G**), which is in keeping with our observations of the lack of changes in follicular reserve or ovarian size in Kiss1r KO mice as function of age (i.e., between 3- and 5-months of age).

### Resting follicle crowding and the probability of initiating growth in the human ovary

To complement our mouse data, we studied whether the relationships between follicle activation and follicle crowding can also apply to the human ovary. Since data for total resting follicle population cannot be obtained because whole ovaries were not available in our archival samples, we analyzed the effects of follicle crowding in the probability of resting follicles to initiate growth and the spatial pattern of distribution in ovaries from cycling women at different ages.

**Fig 3:**
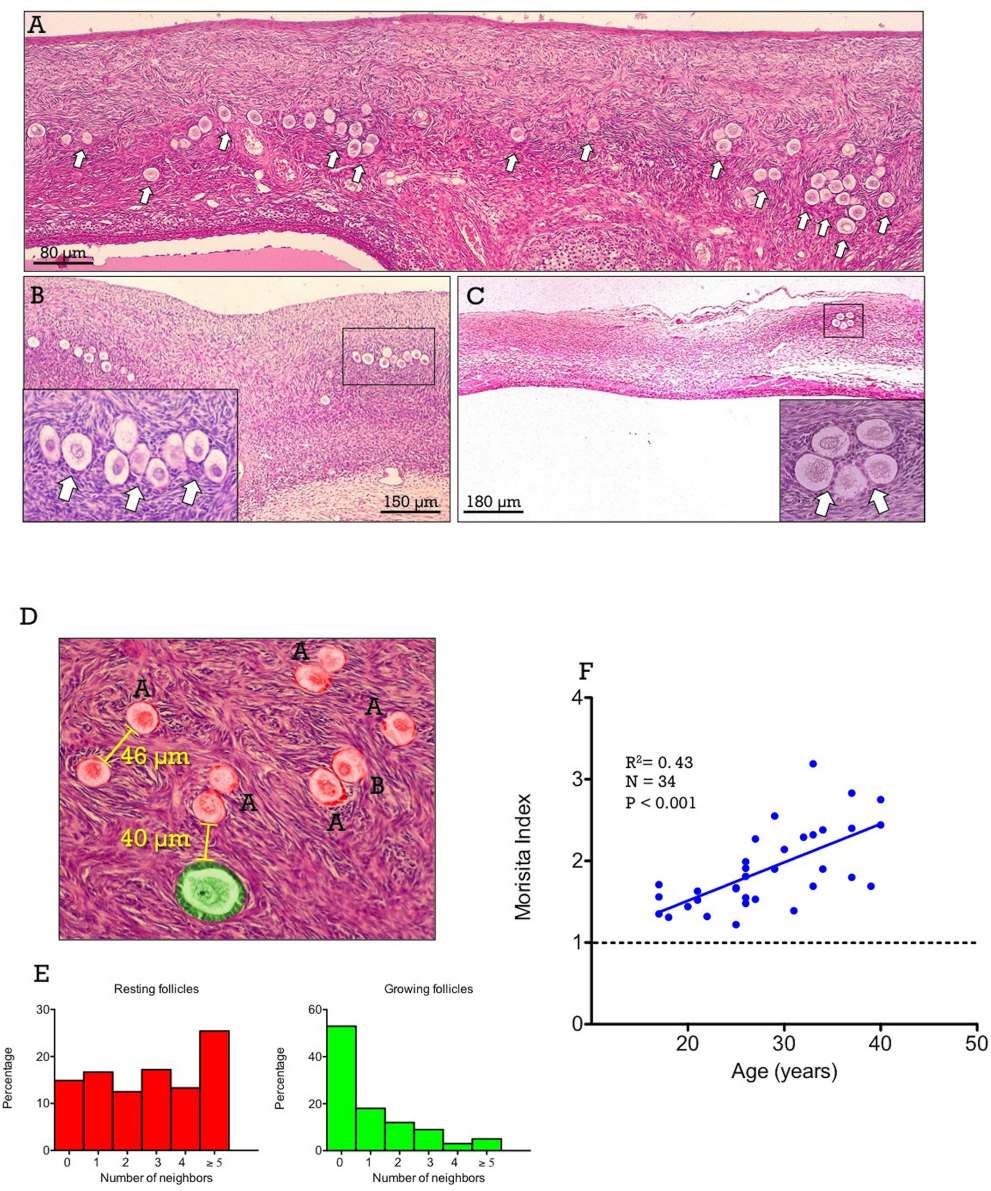
In Panels A-C, representative ovarian sections are presented from 23- (A), 32- (B) and 39- (C) year-old women, showing decreasing density and increasing clustering of resting follicles (arrows) with age. Boxed areas are shown at higher magnification in the insets. In Panel D, a schematic figure is shown illustrating the method used to evaluate the presence of resting follicle neighbors (at a distance ≤ 60-μm) for resting and early growing follicles. In this representative image, one early growing follicle (highlighted in green) that has one neighbor is shown, while several resting follicles are also presented (highlighted in red). Of these resting follicles, those labeled with letter A have one neighbor, whereas those marked with letter B have two neighbors. In Panel E, the distributions of the frequency of the number of neighbors in resting and early growing follicles in human ovaries are presented (pooled data from 20 ovaries). Finally, in Panel F, the correlation between the Morisita’s index of clustering and age in human ovaries is shown. Values above the dotted line indicate clustered spatial pattern.

In the human ovary, due to the larger cortical tissue, the clustered pattern of spatial distribution of resting follicles was even more evident than in the mouse ovary. Representative images of human ovaries from women of different ages, ranging from 23- to 39-years are shown in **Fig 3A–3C**.

To analyze if resting follicle crowding influences the probability of follicles to initiate growth, we determined the number of resting follicle neighbors of both resting and early growing follicles in pooled data from a set of 20 cycling women (ranging age between 20 and 35-years); an example of the method of counting is provided in **Fig 3D**. Our hypothesis was that if resting follicle crowding negatively influences follicle activation, early growing follicles should have less nearby neighbors than dormant follicles. As shown in **Fig 3E**, while most resting follicles (85%) had at least one resting follicle neighbor, the majority (~55%) of early growing follicles lacked resting follicles in their proximity. The existence of a significant association between the presence of neighbors and the follicle status (either resting or growing) was analyzed by calculating the relative probability (RP) and the Odds Ratio (OR) of being growing as function of the presence or not of nearby resting follicle neighbors. As shown in **Table 2**, the probability of being growing is 3.85-times (95% CI: 2.76, 5.35) higher when resting follicle neighbors are absent. On the other hand, the OR indicates that growing follicles have 6.45-times (95% CI: 3.98, 10.44) higher odds of lacking close neighbors. These results are grossly similar to those obtained for the mouse ovary.

**Table 2:** Association analysis between follicle status and the presence of resting follicle neighbors in human ovaries.

	Growing	Resting	RP (95% CI)	OR (95% CI)
Without neighbors	53	58		
With neighbors	47	332	3.85 (2.76–5.36)	6.45 (3.98–10.44)

RP: relative probability of growing; OR: odds ratio

### Age-related changes in resting follicle clustering in the human ovary

The higher probability of isolated resting follicles to initiate growth predicts that resting follicle clustering should increase with age, as non-clustered follicles would leave the resting follicle pool more frequently. To address this quantitatively, we calculated the Morisita’s index of clustering for resting follicles in the total set of human ovaries selected for the study (n = 34; age range between 17- and 40-years). As shown in **Fig 3F**, resting human follicles showed a clustered spatial distribution in all cases, and the degree of clustering showed a strong positive correlation with age.

## Discussion

Regulation of the rate of recruitment of dormant follicles into the growing pool is essential in reproductive biology. This process is thought to be independent of gonadotropic hormones, in contrast to the cyclic recruitment of large pre-antral/early antral follicles, which is gonadotropin-dependent [1,40]. Yet, a role of gonadotropins, particularly FSH, in initial stages of follicle growth has been suggested on the basis of data from hypo-gonadotropic *hpg* mice [41,42]. In our study, we used Kiss1r KO mice as model for sustained (but not complete) hypogonadotropism. Kiss1r KO mice displayed a lower proportion of early growing follicles than WT animals during adult life. This might be due to a role for gonadotropins in initial follicle growth. However, the total number of early growing follicles per ovary was not decreased in Kiss1r KO mice, in agreement with previous studies reporting normal pre-antral follicle growth in mice with disruption of the FSH receptor [43], or in *hpg* mice [44]. Moreover, the possible role of gonadotropins (as systemic, endocrine signals) in follicle activation cannot satisfactorily explain the differential fate of resting follicles in control (WT) mice and women, some of which remain dormant for months or years while others become activated. This differential fate of resting follicles can be better explained by differences in local, micro-environmental conditions.

The data of this study are consistent with the hypothesis that resting follicle crowding is a key factor influencing the rate of recruitment into the growing pool. According to this hypothesis, resting follicles would be the source of local inhibitory signals that decrease the probability of neighboring resting follicles to initiate growth. While the factors responsible for this phenomenon are not exposed by our study, this hypothesis is fully compatible with the pattern of follicle dynamics observed here in post-pubertal mice. According to this model, the presence of higher numbers of other resting follicles in the close vicinity would determine a low rate of recruitment, thus preventing premature exhaustion of the ovarian follicle reserve. On the other hand, the age-mediated decrease in follicle crowding, as consequence of previous follicular recruitment and attrition, would favor an increase in the rate of recruitment, thus providing enough number of growing follicles to maintain ovulation up to advanced ages, even when the ovarian reserve is highly depleted. Finally, differences in follicle crowding, which depend to a large extent on differences in the spatial pattern of clustering, would provide a mechanism for the differential probability of any given resting follicle to initiate growth. In this context, the striking uneven distribution of resting follicles in the ovarian cortex, particularly evident in the human ovary [45], would constitute the structural counterpart of the humoral (local, endocrine) mechanism regulating follicle activation.

Even young (1-mo-old) pre-pubertal mice showed a clustered spatial distribution of resting follicles; an observation that is line with data from young peri-pubertal girls, who show uneven distribution of primordial follicles [45]. Thus, although our data document that primordial follicle clustering increases with age, some degree of clustering is already established during pre-pubertal development, when large numbers of primordial follicles are lost by oocyte death in rodent [10,12] and human [11] ovaries. Whether follicle attrition follows a particular spatial pattern is not yet known.

Our results are roughly in agreement with the hypothesis of Da Silva-Buttkus and co-workers [22], based on histological analyses done only in neonatal mice. It must be stressed, however, that during the first postnatal days, virtually all growing-follicles belong to the pool of medullary (not cortical) follicles, thus limiting the value of observations at this early window on the prediction of follicular dynamics later in postnatal life. As methodological note, in our study, we considered inter-follicular distances ranging from 40-μm (for mouse resting follicles) to 60-μm (for mouse early growing follicles and human small follicles) to defining neighboring follicles. Such distances are in general larger than those defined in the study by Da Silva-Buttkus and co-workers [22], in which the largest distance at which a neighbor effect was found in the neonatal ovary was actually 40-μm. Those distance threshold, though, were considered appropriated for our study, considering that in the postnatal ovary resting follicles are not so densely packed as in neonatal ovaries. In addition, we did not assess the possible influence of the proximity to the ovarian surface epithelium, since the distance between resting follicles and ovarian surface was considerable large in human ovaries.

Our model proposing a role of resting follicle crowding in follicle activation in adulthood raises several predictions of potential interest in translational reproductive medicine. Conditions involving decreased ovarian volume, without a concomitant decrease of the ovarian reserve, would result in increased follicle crowding and, consequently, a reduced rate of recruitment of resting follicles. In good agreement, a decreased loss of resting follicles is observed in hypophysectomized rodents [46], rats treated with GnRH-antagonists [47], and in hpg mice [44]. Likewise, our present data document that Kiss1r KO mice display also an enlargement of the resting follicle pool, despite later arrest of folliculogenesis and anovulation [48]. In contrast, conditions involving enlargement of ovarian volume would decrease follicle crowding, presumably leading to a higher rate of recruitment. This is in keeping with the observed increase in follicle activation in some studies after treatment of *hpg* mice with gonadotropins [41,42,44], which causes an increase in ovarian volume due to the development of large antral follicles and (eventually) corpora lutea. On the other hand, according to the model proposed here, a decrease in the ovarian reserve would exacerbate follicle loss. Both observational and experimental studies support this prediction. An accelerated loss of resting follicles has been reported in pre-menopausal women, from 37-yrs of age onwards [49], coincident with the decrease in the ovarian reserve. In rats, busulphan-induced depletion of primordial follicles determines an increased recruitment of resting follicles [21]. In that study, when the pool of resting follicles was highly decreased, the ovarian reserve was rapidly exhausted, suggesting that depletion of resting follicles is a self-exacerbating condition. All in all, the above observations point out the existence of a threshold in follicle density that accelerates follicle activation. In good agreement, we observed a large increase in the proportion of early growing follicles when the ovarian reserve fell below ~500 resting follicles per ovary.

Follicle activation has been proposed to be negatively regulated by growing follicles, and experimental data supports the existence of inhibitory signals derived from mid-growing follicles, such as anti-mullerian hormone, AMH [50], acting in an endocrine and/or paracrine manner. AMH produced by growing follicles inhibits primordial follicle activation, therefore operating as an inter-follicular negative feedback mechanism [40]. Thus, AMH has been considered to be an indirect reliable marker of the existing ovarian reserve. However, the lower rate of recruitment in Kiss1r KO mice, which lack antral follicles and have decreased numbers of large secondary follicles, is difficult to reconcile with the idea that the number of growing follicle is the only factor regulating follicle activation. In any event, it is stressed that the existence of inhibitory actions of factors derived from growing follicles is not in contradiction (but rather fully compatible) with the resting follicle-*crowding* hypothesis proposed herein.

A problem when (morphologically) studying follicle activation is that the limits to define when a follicle is actually growing are complex to set. Transition from primordial to primary follicular stage is a complex multi-step protracted process, involving changes in both the oocyte and surrounding somatic cells. Pre-granulosa cells change from flattened to cuboidal and proliferate increasing cell numbers at the same time that the oocyte initiates growth. Different criteria have been used to classify small follicles as resting or growing. In this study, we have used a final-step phase, by considering as growing those follicles that show evident signs of activation, such as increased numbers of granulosa cells and oocyte growth. According to this criterion, primordial, transitional and early primary follicles (lacking oocyte growth and with follicle size >30-μm in diameter) were globally considered as resting follicles. Similar criteria, defined for type 3b follicles according to Pedersen & Peters classification [26], have been used in previous studies, in both rodents [27] and humans [28–30]. Likely, the different steps mediating the transformation of primordial (clearly resting) into large primary (clearly growing) follicles require different combinations of inhibitory and stimulatory factors.

The age-related decrease in the ovarian reserve is due to both recruitment of resting follicles into the growing pool and resting follicle attrition. Follicle death was not considered in our study, because resting follicle death is rarely observed in ovarian sections from adult animals, as dying follicles are rapidly eliminated. Previous studies suggest that follicle attrition is abundant in neonatal and pre-pubertal ovaries [11,12], but that follicle recruitment into the growing pool seems to be the main cause of resting follicle loss in physiological conditions in post-pubertal ovaries. However, the great majority of these growing follicles undergo atresia after activation, mainly at advanced (i.e., early antral) stages. The mechanisms regulating primordial follicle survival and the interrelationship between survival and activation remain poorly understood [13]. The persistence of high numbers of resting follicles in Kiss1r KO mice would suggest that gonadotropins are not essential surviving factors for primordial follicles. In any event, it is stressed that our present model proposes that the rate of recruitment depends on spatial (crowding) determinants, as defined by the size and spatial pattern distribution of the resting follicular pool, irrespective of whether changes in the ovarian reserve are due to follicle attrition and/or previous recruitment into the growing pool.

As note of caution, we used in our study female Kiss1r KO mice as model of sustained hypo-gonadotropism. While this model suffers infertility of central origin, and gonadotropin levels are significantly suppressed, LH and FSH are clearly detectable in circulation. In fact, whereas circulating FSH levels are markedly decreased, LH concentrations are only partially lowered in Kiss1r KO mice [23]; hence, some degree of gonadotropic drive is preserved in our model, which is, nonetheless, clearly insufficient to promote full follicular growth and ovulation [48]. On the other hand, local expression and actions of the kisspeptin system in the mammalian ovary have been documented by different groups, including ours [48,51–54]; indeed, insufficient kisspeptin signaling in the oocyte has been recently proposed as potential mechanisms for premature ovarian insufficiency (POI) in rodents. Hence, while results concerning resting follicle reserve and activation in Kiss1r KO mice are compatible with our *crowding* hypothesis, our data do not at all exclude local actions of kisspeptins in the ovary.

In addition, it is noted that a limitation of our study is that only bi-dimensional relationships were analyzed, instead of tri-dimensional analysis, which would require complex computer-assisted image analysis systems that are not currently available at our group. Nonetheless, we would like to stress that the lack of tri-dimensional analysis is not a differential bias (between groups) nor we consider that it invalidates our major conclusions. In fact, a recent tri-dimensional study, using fluorescence–driven whole organ analysis, reported a clustered distribution of oocytes in mouse ovaries [31]. Indeed, the overall similarities between the results of this 3D imaging analysis and the current data reinforce the validity our 2D analysis. It must be stressed, though, that our study provides novel information regarding the influence of resting follicle neighbors on the probability to initiate growth in the ovaries of mice (including a model of hypogonadotropism) and humans, which was not included in the former methodological 3D analysis.

In sum, we propose here that spatial features, as defined by the size, pattern of tissue distribution and clustering, of the resting follicle pool are major determinants of the fate of individual resting follicles, i.e., to become activated or remain dormant, thus playing a significant role in the maintenance of the follicular reserve and providing the structural/morphological counterpart to the humoral signals involved in activation/repression of early follicular growth. Such crowding-mediated influence on follicle activation has potential pathophysiological implications in ovarian ageing and disease. For instance, decreased ovarian reserve or follicular density would accelerate follicle reserve depletion, leading to POI. On the other hand, differences in ovarian volume, resting follicle density and initial recruitment have been reported in patients with polycystic ovarian disease [55,56]. Whether alterations in resting follicle crowding and spatial pattern distribution do occur in these conditions remains unexplored.

## Supporting Information

S1 Fig Scheme of the method applied to obtain the Morisita’s index of clustering in the human ovary. A square of 400 x 400 μm was displaced in consecutive fields and the number of resting follicles in each square unit recorded. Follicles that are intercepted by the black line are not counted, whereas those intercepted by the yellow line are considered in the field. In the example, the numbers of follicles in the 5 square units are: 0, 6, 1, 3, and 7 (denoted by arrows). A total of 50 square units per ovary were scored.
(PDF)

S1 File Representative images are presented of resting and early growing follicles in the mouse (Panel A) and human (Panel B) ovary, showing primordial (P), transitional (T), small primary (SPr), late primary (LPr) and small secondary (SSF) follicles. (PDF)
