# Supplementary material for: Crowding and Follicular Fate: Spatial Determinants of Follicular Reserve and Activation of Follicular Growth in the Mammalian Ovary
Source: PLoS One. 2015 Dec 7;10(12):e0144099. doi: 10.1371/journal.pone.0144099 (PMC4671646; doi:10.1371/journal.pone.0144099)
Supplement: S1 Fig — A square of 400 x 400 μm was displaced in consecutive fields and the number of resting follicles in each square unit recorded. Follicles that are intercepted by the black line are not counted, whereas those intercepted by the yellow line are considered in the field. In the example, the numbers of follicles in the 5 square units are: 0, 6, 1, 3, and 7 (denoted by arrows). A total of 50 square units per ovary were scored. (PDF) [file pone.0144099.s001.pdf]

Human Ovary

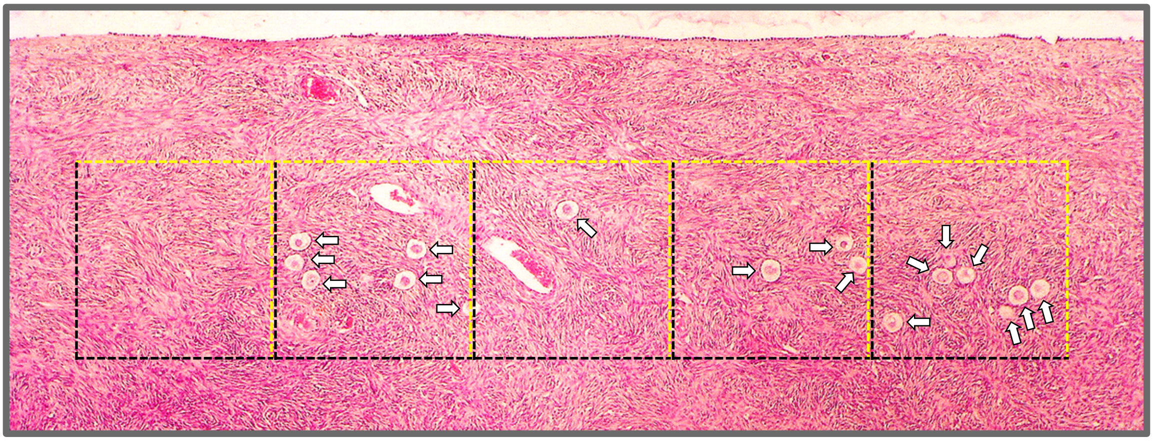

S1 Fig: Scheme of the method applied to obtain the Morisita's index of clustering in the human ovary. A square of 400 x 400 µm was displaced in consecutive fields and the number of resting follicles in each square unit recorded. Follicles that are intercepted by the black line are not counted, whereas those intercepted by the yellow line are considered in the field. In the example, the numbers of follicles in the 5 square units are: 0, 6, 1, 3, and 7 (denoted by arrows). A total of 50 square units per ovary were scored.
