# Supplementary material for: Crowding and Follicular Fate: Spatial Determinants of Follicular Reserve and Activation of Follicular Growth in the Mammalian Ovary
Source: PLoS One. 2015 Dec 7;10(12):e0144099. doi: 10.1371/journal.pone.0144099 (PMC4671646; doi:10.1371/journal.pone.0144099)
Supplement: S1 File — (PDF) [file pone.0144099.s002.pdf]

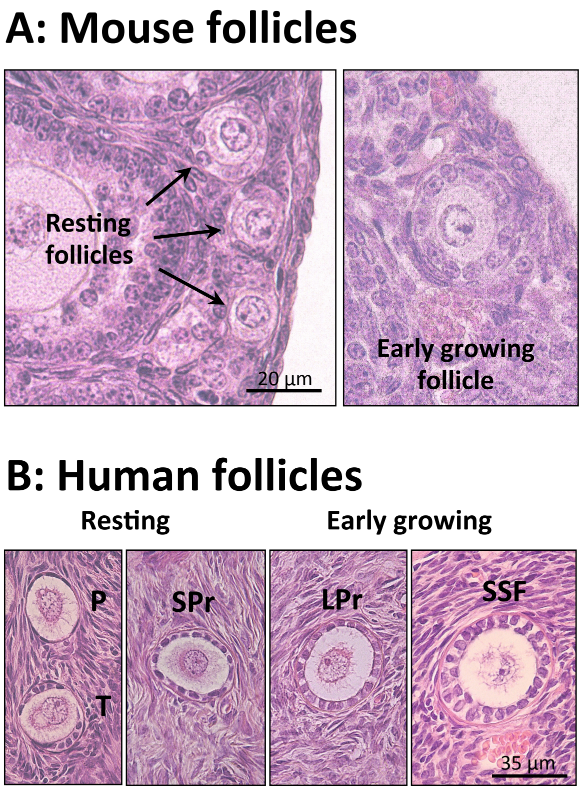

S1 File: Representative images are presented of resting and early growing follicles in the mouse (**Panel A**) and human (**Panel B**) ovary, showing primordial (P), transitional (T), small primary (SPr), late primary (LPr) and small secondary (SSF) follicles.
